# Evaluation of Intracranial Vasculatures in Healthy Subjects with Arterial-Spin-Labeling-Based 4D-MR Angiography at 3T

**DOI:** 10.2463/mrms.tn.2015-0081

**Published:** 2015-12-22

**Authors:** Yasuhiko IRYO, Toshinori HIRAI, Masanobu NAKAMURA, Machiko TATEISHI, Eri HAYASHIDA, Minako AZUMA, Shinichiro NISHIMURA, Mika KITAJIMA, Yasuyuki YAMASHITA

**Affiliations:** 1 Department of Diagnostic Radiology, Graduate School of Medical Sciences, Kumamoto University 1-1-1, Honjo, Kumamoto 860-8556, Japan; 2 Department of Radiology, University of Miyazaki; 3 Philips Electronics Japan

**Keywords:** arterial spin labeling, MR angiograpy, healthy subjects, intracranial vasculatures, 3T

## Abstract

Contrast inherent inflow-enhanced multi-phase angiography combining multiple-phase flow-alternating inversion-recovery (CINEMA-FAIR) is an arterial-spin-labeling-based four-dimensional magnetic resonance angiography (4D-MRA) technique. Two neuroradiologists independently evaluated the depiction of the intracranial vasculatures in healthy subjects with 3T 4D-MRA using CINEMA-FAIR. Our results indicated that this technique can provide good visualization of the cerebral arteries with a high spatial and temporal resolution. It appears to have sufficient resolution for identifying flow difference in the anterior and posterior circulation in healthy subjects.

## Introduction

The evaluation of anatomic and hemodynamic characteristics of cerebral vascular disease, such as carotid artery steno-occlusive disease, intracranial arteriovenous malformations (AVMs), and intracranial dural arteriovenous fistulae (DAVF), are important for accurate diagnosis, effective treatment, and follow-up examination.^1,2^ Intra-arterial digital subtraction angiography (DSA) remains the gold standard for the assessment of their cerebral vascular disease. However, DSA is invasive, exposes the patient to radiation, and requires the injection of iodinated contrast material.^3^

Four-dimensional (4D) contrast-enhanced magnetic resonance angiography (MRA) at 3T appears to be reliable and provides hemodynamic information on cerebral vascular disease^4^ However, it poses risks associated with contrast agents.^5^

Recently, one of the promising methods without using radiation and exogenous contrast agents for evaluating anatomic and hemodynamic characteristics of cerebral vascular diseases is arterial-spin-labeling (ASL)-based 4D-MRA.^6–8^ Similar to DSA, this technique makes the sequential observation of hemodynamics possible and it facilitates large 3D-volume acquisitions. Among the ASL-based 4D-MRA techniques, contrast inherent inflow-enhanced multi-phase angiography combining multiple-phase flow-alternating inversion-recovery (CINEMA-FAIR) can provide anatomic and hemodynamic information of intracranial vasculatures with high spatial and temporal resolution in clinical subjects, such as patients with dural arteriovenous fistulas^6^ or carotid artery steno-occlusive disease.^9^ To our knowledge, however, it has not been fully investigated how the intracranial arteries in healthy subjects are visualized on 4D-MRA using CINEMA-FAIR. It is important for evaluating various diseases with this technique to establish reference data obtained from healthy subjects. The aim of this study was to evaluate the visualization of the intracranial vasculature in healthy subjects with 3T 4D-MRA using CINEMA-FAIR.

## Materials and Methods

### Study population

Our study was approved by our institutional review board; prior informed consent for the imaging studies was obtained from all volunteers. All studies were performed on a 3T MR imaging system (Achieva, Philips Medical Systems, Best, The Netherlands). Thirteen healthy volunteers (2 women, 11 men; age 24–82 years, mean 51.9 years) underwent the ASL-based 4D-MRA study. Inclusion criteria were no past history of cardiovascular disorders, and no contraindications, such as claustrophobia or pregnancy, for MR examination.

### ASL-based 4D-MRA technique

All MR studies were performed on a 3T MRI scanner (Achieva, Philips Medical Systems) using a commercially available 32-channel head coil. The MRI unit featured a gradient system with maximal achievable gradient amplitude of 40 mT/m and a slew rate of 200 T/m/s.

For ASL-based 4D-MRA imaging, we used CINEMA-FAIR; details on the sequence are available elsewhere.^6^ This MR technique combines ASL with a 3D segmented T_1_-weighted turbo field echo sequence (3D-T_1_ TFE). The FAIR preparation scheme with look-locker sampling was used for CINEMA-FAIR. Each sequence used for the measurements consisted of two acquisitions with identical readouts and different magnetization preparation schemes. Imaging data were from two consecutive acquisitions preceded by nonselective- and spatially selective inversion pulses. After completion of the two acquisitions, the corresponding temporal phases from two acquisitions with identical inversion delays were subtracted.

The parameters for the CINEMA-FAIR sequence were (repetition time) TR, 4.5 ms; (echo time) TE, 2.2 ms; flip angle, 10°; field of view (FOV), 200 × 200 mm^2^; matrix, 192 × 192; slice thickness, 1 mm; slab thickness, 100 mm; reconstructed spatial resolution, 0.5 × 0.5 × 0.6 mm^3^; sensitivity encoding (SENSE) factor, 3.0; and acquisition time, 8 m 26 s. Images of dynamic inflow were obtained with successive acquisitions with labeling delay times of 80, 380, 680, 980, 1280, 1580, and 1880 ms.

### Image analysis

Two readers (M.A. and T.H. with 7 and 24 years of experience in neuroradiologic imaging, respectively) independently evaluated the ASL-based 4D-MRA images on a picture-archiving and communication system (PACS) workstation. The 4D data were displayed with all regions visible. The software allowed the enlargement of regions of special interest in any given spatial orientation.

ASL-based 4D-MRA data were assessed with respect to overall image quality, arterial/venous separation, arterial vascular visualization, and differences in the visualization of the internal carotid artery (ICA) and vertebrobasilar artery (VBA). With regard to arterial vascular visualization, they evaluated the following nine arterial segments on ASL-based 4D-MRA images: the ICA, middle cerebral artery (MCA: M1, M2–M3, and M4 segments), anterior cerebral artery (ACA: A1–A2 and A3–A4 segments), posterior cerebral artery (PCA: P1–P2 and P3–P4 segments), and basilar artery (BA).

Using a 4-point grading system, they first scored the overall image quality, where grade 1 = nondiagnostic due to severe artifacts, grade 2 = artifacts that may interfere with the diagnosis, grade 3 = artifacts that do not interfere with the diagnosis, and grade 4 = diagnostic without artifacts. Then they scored arterial/venous separation, where grade 1 = extensive mixing of venous and arterial signal intensity (SI), grades 2 and 3 = moderate and slight contamination of venous SI, respectively, and grade 4 = arterial SI only. They also scored the arterial vascular visualization, where grade 1 = insufficient vessel depiction or blurring of the vessel contours, grade 2 = suboptimal arterial depiction for confident diagnosis, grade 3 = relatively adequate depiction for confident diagnosis, and grade 4 = sufficient vessel depiction with confident diagnosis. Finally, they recorded differences in the early visualization of the top portion of the ICA and VBA, where grade 1 = visualization of the top portion of the ICA precedes that of the VBA by about 2 phases (600 ms), grade 2 = visualization of the top portion of the ICA precedes that of the VBA by about 1 phase (300 ms), grade 3 = visualization of the top portion of the ICA precedes that of the VBA by less than 1 phase (300 ms), and grade 4 = no apparent difference. In addition, with regard to the differences in the visualization of the ICA and VBA on ASL-based 4D-MRA images, volunteer subjects were divided into two groups including 20–50 year olds and 51–80 year olds to understand the effect of age, and they were also evaluated.

### Statistical analysis

Interobserver agreement for ASL-based 4D-MRA images with respect to overall image quality, arterial/venous separation, arterial vascular visualization, and differences in the visualization of the ICA and VBA was determined by calculating the κ coefficient (κ < 0.20, poor-; κ = 0.21–0.40, fair-; κ = 0.40–0.60, moderate-; κ = 0.61–0.80, good-; κ = 0.81–0.90, very good-; and κ > 0.90, excellent agreement) including the 95% confidence interval (CI). A statistical package, Med-Calc for Windows (MedCalc Software, Mariakerke, Belgium), was used for all analyses.

## Results

**Fig. 1.**
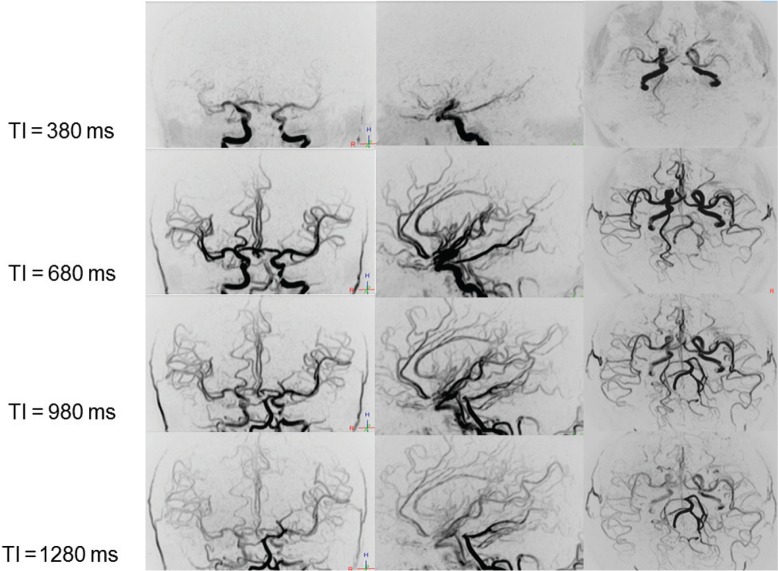
Arterial-spin-labeling (ASL)-based four-dimensional magnetic resonance angiography (4D-MRA) images in a 30-year-old healthy male volunteer. Anteroposterior (left), lateral (middle), and axial (right) projections of maximum intensity projection images acquired at 300 ms temporal- and 0.5 × 0.5 × 0.5 mm^3^ spatial resolution. ASL-based 4D-MRA images show intracranial arteries without contamination of venous structures as well as distal cerebral arteries with uniform signal intensity. Depiction of the top portion of the internal carotid arteries (ICAs) precedes that of the vertebrobasilar arteries (VBAs) by about 1 phase (300 ms). On the basis of these findings, both observers judged that the overall image quality, the arterial/venous separations were grade 4 and that the difference in the early visualization between the top portions of the ICA and the VBA was grade 2. Both readers assigned grade 4 for visualization of all arterial vascular segments. T_I_, inversion time

Both readers judged the overall image quality on ASL-based 4D-MRA images of all the 13 volunteers as diagnostic without artifacts (grade 4) (Fig. 1). Arterial/venous separation was scored as grade 4 in 12- and grade 3 in 1 volunteer for both readers, respectively. Interobserver agreement was excellent (κ = 1.0).

In the M1, M2–3, A1–2, P1–2, and BA segments, the arterial vascular visualization were scored as grade 4 in all the 13 volunteers for both readers. Although the mean score of arterial vascular visualization tended to slightly lower in the ICA, M4, A3–4, and P3–4 segments than previously mentioned segments, the mean score of their segments were more than 3.7 for both readers (ICA: 3.76 [both readers]; M4: 3.92 [reader 1], 3.76 [reader 2]; A3–4: 3.76 [both readers]; P3–4: 3.92 [both readers]). Interobserver agreement was good (κ = 0.76; 95% CI, 0.528–1.0) (Table 1).

Differences in the early visualization of the top portion of the ICA and VBA were assigned a score of grade 2 in 9- and of grade 3 in 4 volunteers by reader 1; reader 2 was assigned grade 2 in 7- and grade 3 in 6 volunteers (Fig. 1). Interobserver agreement was good (κ = 0.683; 95% CI, 0.279–1.0) (Table 2). When volunteer subjects were divided into two groups including 20–50 year olds and 51–80 year olds, the difference in the visualization of the ICA and VBA for the elder groups tended to be greater than for the younger groups (Table 3).

## Discussion

4D-MRA using CINEMA-FAIR covered the whole brain with a high spatial (0.5 × 0.5 × 0.6 mm^3^) and temporal resolution (300 ms) with diagnostic quality in all healthy subjects. Intracranial arteries were visualized without contamination of venous structures in almost cases. ASL-based 4D-MRA images were able to depict not only the proximal cerebral arteries but also the distal cerebral arteries. These results may be attributed to a high signal-to-noise ratio (SNR) from use of a 3T MR unit and a 32-channel head coil.

Arterial/venous separation was scored as slight contamination of venous SI (grade 3) in one case. In this case, the left sigmoid to inferior petrous sinus was depicted on ASL-based 4D-MRA image. On a previous study on 3T time-of-flight (TOF) MRA,^10^ this finding may be the result of flow reversal. The other venous structures including the superior sagittal sinus were not depicted on ASL-based 4D-MRA images. We think that this result attributes to the use of a saturation pulse in the cranial region of the FOV. In a few cases, the arterial vascular visualization was not scored as sufficient vessel depiction with confident diagnosis (grade 4) but relatively adequate depiction for confident diagnosis (grade 3) in proximal arterial segment, such as ICA. These results may be attributed to atherosclerosis in aged subjects.

In our study, the visualization of the intracranial arteries was earlier for the top portion of the ICA than the VBA. A previous ultrasound study on the blood flow velocity in extracranial arteries^11^ showed a flow difference between the ICA and vertebral artery (VA). The peak systolic velocity (PSV) in the ICA was 76 ± 14 cm/s in individuals aged between 20 and 50 years and 65 ± 14 cm/s in those aged between 51 and 80 years. On the other hand, PSV in the VA was 53 ± 10 cm/s in the former and 48 ± 12 cm/s in the latter age group. In both age groups, PSV was approximately 20 cm/s faster for the ICA than the VA.^11^ In addition, a previous study using an ASL-based MRA technique show different blood arrival times of each intracranial vascular segment in volunteers.^12^ The mean relative transit time of the blood (rTT) was 173.7 ± 51 ms for the ICA and 469.4 ± 129 ms for the VA. The mean rTT was about 300 ms earlier for the ICA than the VA. Our study showed 4D-MRA with CINEMA-FAIR appears to have sufficient resolution for detecting the flow difference in the anterior and posterior circulation. In addition, the difference in the visualization of the ICA and VBA for the elder groups tended to be greater than for the younger groups. A previous study^11^ stated that there were significant decrease in blood flow velocity in the ICA and VA related to increased age.

Other time-resolved ASL-based MRA techniques using FAIR at 3T (e.g., true fast imaging with steady-state precession-based spin tagging with alternating radiofrequency [True STAR] technique) have been reported.^7^ This True STAR technique uses the balanced steady-state free precession (bSSFP) for data acquisition. Compared to CINEMA-FAIR using T_1_-TFE sequences such as the fast low angle shot, the bSSFP readout is generally more prone to off-resonance artifacts. Such artifacts may be more prevalent when imaging at higher field strength. In addition, CINEMA-FAIR technique has wide labeling area, theoretically resulting in an increase of the signal from flowing blood on the slice.

Our study has some limitations. First, we did not compare ASL-based 4D-MRA and DSA findings. As DSA is an invasive technique, it could not be applied for volunteers. Second, ours was a single-center study and the study population was small. Third, we did not evaluate the PSV with ultrasound examination.

## Conclusion

On 3T 4D-MRA images using CINEMA-FAIR the intracranial arteries in healthy volunteers were visualized at a high spatial and temporal resolution without exogenous contrast agents. This technique appears to have sufficient temporal resolution for the identification of flow difference in the anterior and posterior circulation. The flow difference in the anterior and posterior circulation should not be considered abnormal for assessing various intracranial vascular diseases.

## Figures and Tables

**Table 1.** Interobserver agreement for the arterial vascular visualization

	4D-ASL MRA		Interobserver agreement*

	Observer 1	Observer 2	
Grade 1	0	0	
Grade 2	0	0	0.76
Grade 3	8	10	(0.528–1.0)
Grade 4	109	107	

4D-ASL MRA, four-dimensional arterial spin labeling magnetic resonance angiography; Grade 1, insufficient vessel depiction or blurring of the vessel contours; Grade 2, suboptimal arterial depiction for confident diagnosis; Grade 3, relatively adequate depiction for confident diagnosis; Grade 4, sufficient vessel depiction with confident diagnosis

* Data are k statistics, and numbers in parentheses are 95% confidence intervals.

**Table 2.** Interobserver agreement for the differences in the early visualization of the interanal carotid artery (ICA) and vertebrobasilar artery (VBA)

	4D-ASL MRA		Interobserver agreement*

	Observer 1	Observer 2	
Grade 1	0	0	
Grade 2	9	7	0.683
Grade 3	4	6	(0.279–1.0)
Grade 4	0	0	

4D-ASL MRA, four-dimensional arterial spin labeling magnetic resonance angiography; Grade 1, visualization of the ICA precedes that of the VBA by about 2 phases (600 ms); Grade 2, visualization of the ICA precedes that of the VBA by about 1 phase (300 ms); Grade 3, visualization of the ICA precedes that of the VBA by less than 1 phase (300 ms); Grade 4, no apparent difference

* Data are k statistics, and numbers in parentheses are 95% confidence intervals.

**Table 3.** Differences in the early visualization of the interanal carotid artery (ICA) and vertebrobasilar artery (VBA) of different age groups

(Age range, years)	Observer 1		Observer 2	
	21–50	51–80	21–50	51–80
Grade 1	0	0	0	0
Grade 2	4	5	2	5
Grade 3	3	1	5	1
Grade 4	0	0	0	0

4D-ASL MRA, four-dimensional arterial spin labeling magnetic resonance angiography; Grade 1, visualization of the ICA precedes that of the VBA by about 2 phases (600 ms); Grade 2, visualization of the ICA precedes that of the VBA by about 1 phase (300 ms); Grade 3, visualization of the ICA precedes that of the VBA by less than 1 phase (300 ms); Grade 4, no apparent difference
